# Effect of Processing Parameters on the Creep Behavior and Integrity of Plasma-Sprayed Thermal Barrier Coatings on Ti-6Al-4V

**DOI:** 10.3390/ma19101958

**Published:** 2026-05-09

**Authors:** Bianca Costa Rodrigues, Renata Jesuina Takahashi, Vera Lúcia Othéro de Brito, Danieli Aparecida Pereira Reis

**Affiliations:** 1 Laboratory of Mechanical Behavior of Metals (LCMM), Institute of Science and Technology, Federal University of São Paulo (UNIFESP), São José dos Campos 12231-280, SP, Brazil; bianca.costa@unifesp.br (B.C.R.); renata.takahashi@unifesp.br (R.J.T.); 2 Institute for Advanced Studies (IEAv), Department of Science and Aerospace Technology, São José dos Campos 12228-001, SP, Brazil; veravlob@fab.mil.br

**Keywords:** thermal barrier coatings, creep behavior, scratch testing, plasma spraying, Ti-6Al-4V alloy

## Abstract

**Highlights:**

**What are the main findings?**

- APS parameters controlled TBC microstructure, crack formation, porosity, and surface integrity.
- Higher porosity and pre-existing microcracks increased creep rate (167% higher) and damage.
- Lower porosity and crack-free microstructure reduced creep rate (~67% lower), and delayed damage evolution.
- Scratch testing and instrumented indentation revealed higher compliance and stress redistribution in thin TBC.
- Coatings affected creep damage kinetics without altering substrate fracture behavior.

**What are the implications of the main findings?**

- Creep resistance of coated Ti-6Al-4V depends on microstructure and stress distribution.
- Mechanical compliance improves stress redistribution during creep.
- APS processing parameters must be tailored to avoid premature cracking.
- Results support TBC design for high-temperature aerospace applications.

**Abstract:**

This study investigates how processing parameters and powder characteristics influence the mechanical performance of thermal barrier coatings (TBCs) applied to a Ti-6Al-4V alloy. Two TBCs were deposited by Atmospheric Plasma Spray (APS) using different processing conditions, feedstock characteristics, and coating thicknesses (thin and thick configurations). TBC characterization included powder size analysis, scanning electron microscopy (SEM), surface roughness, X-ray diffraction, instrumented indentation, and scratch testing. Mechanical behavior was assessed using creep testing at 125 MPa and 500 °C for coated and uncoated samples. Fracture surfaces of crept samples were analyzed by SEM and stereomicroscopy. Thicker TBC exhibited higher elastic modulus but contained microcracks and higher porosity, resulting in a higher steady-state creep rate (0.0006 h^−1^, approximately 167% above the uncoated substrate) and reduced rupture time. Conversely, thinner TBC remained initially crack-free, promoting stress redistribution and leading to a lower creep rate (0.0002 h^−1^, about 67% below the substrate) and delayed failure. Fractographic analysis revealed ductile fracture of Ti-6Al-4V in all conditions, indicating that coatings influenced damage accumulation rather than fracture mode. These findings underscore the combined effect of processing parameters and coating architecture on TBC performance for aerospace applications.

## 1. Introduction

**Figure 1:**
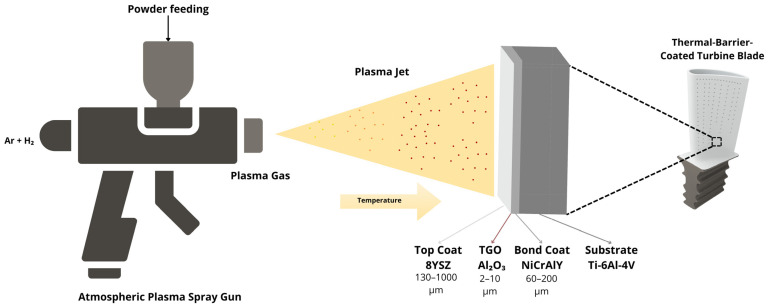
Schematic representation of the Atmospheric Plasma Spray (APS) process used for depositing thermal barrier coatings in aerospace applications.

Thermal barrier coatings (TBCs) are critical for protecting metallic components operating under severe thermal and mechanical conditions in aerospace applications. A typical TBC system consists of a substrate, a metallic bond coat (BC), a thermally grown oxide (TGO) layer formed by BC oxidation, and a ceramic top coat (TC). Each layer exhibits distinct physical and chemical properties, but the deposition process has key relevance in determining coating adhesion and overall performance [1,2]. Among the available deposition processes, atmospheric plasma spray (APS) (Figure 1) remains one of the most widely adopted industrial techniques due to its applicability to a wide range of materials, relatively simple operation, significant insulation performance and ability to produce coatings with controlled thickness [3,4].

Studies have shown that processing parameters and initial powder characteristics strongly influence the splat morphology, interlamellar bonding, and overall microstructure of APS coatings, which ultimately govern their mechanical and thermal performance [5,6]. One particularly key variable affecting coating performance is thickness. TBCs deposited via APS typically have top coat thicknesses ranging from approximately 130 μm up to 1 mm, depending on the required thermal protection level and component geometry. For aeroengine applications, coatings are generally maintained below 500 μm, whereas thicker coatings have been proposed for more demanding environments, such as hypersonic systems [7]. Particularly, top coat thicknesses approaching 1 mm have been reported in so-called super-thick thermal barrier coatings (STTBCs), representing the upper range of thickness explored in APS systems [7]. Conversely, thin TBCs, typically defined in engine-related applications as coatings with thicknesses below approximately 250 μm, have been proposed as an alternative approach due to their reduced thermal mass and improved thermomechanical response [8]. These variations in thickness are associated with distinct residual stress states, microstructural features, and damage mechanisms that directly affect coating performance. While thicker coatings offer superior thermal insulation, they also introduce higher residual stresses and more pronounced microstructural gradients. Recent studies, including Man et al., have shown that the interplay between deposition parameters, splat microstructure, and thickness significantly influences creep behavior, stress-assisted crack propagation, and failure mechanisms in multilayer ceramic coatings [7]. Conversely, early investigations have suggested that thinner TBCs could still provide meaningful thermal protection while minimizing thermal mismatch stresses and weight, which is particularly advantageous for components subjected to strict mechanical constraints, such as engine applications [8]. Thus, understanding the mechanical implications of coating thickness, especially under time-dependent high-temperature deformation, is crucial for ensuring long-term reliability.

Although less commonly associated with TBC systems than nickel-based superalloys, titanium alloys like Ti-6Al-4V are increasingly relevant for aerospace applications where weight reduction and high specific strength are critical. Ti-6Al-4V is widely used due to its excellent strength-to-weight ratio, corrosion resistance, and good mechanical performance at intermediate temperatures [9]. However, its application at elevated temperatures is limited by a significant reduction in strength and dimensional stability above 500 °C due to oxidation and creep initiation, which is a time-dependent phenomenon where the material experiences slow and continuous plastic deformation at elevated temperatures (above 0.4 of the melting temperature) [10,11]. Under such conditions, Ti-6Al-4V components may operate close to their thermal stability limit, in which time-dependent deformation and environmental degradation become critical factors. In this context, recent studies have shown that the creep behavior of Ti-6Al-4V is strongly governed by microstructural features and deformation mechanisms, particularly involving dislocation glide and climb, which are influenced by phase distribution and lamellar morphology [9].

Surface modification techniques, such as plasma-based ion implantation, have been shown to influence the creep behavior of Ti-6Al-4V alloys at elevated temperatures [12], but these methods are less cost-effective and less commonly used than coating approaches. These limitations motivate TBCs application, which represent a promising strategy to reduce thermal exposure, delay oxidation processes, and extend component lifetime [1,4]. However, only a limited number of studies have systematically investigated the combined effects of APS processing parameters, powder characteristics, and coating thickness on the creep performance of TBC systems applied to titanium alloys. Particularly, the specific role of APS parameters in controlling microstructural features such as microcrack formation, interlamellar bonding, and stress distribution under creep conditions remains insufficiently understood.

Given this context, this work investigates the influence of processing parameters, powder characteristics, and coating thicknesses on the microstructure, integrity and mechanical performance of plasma-sprayed TBCs applied to Ti-6Al-4V. We produced two distinct coatings using industrial APS conditions: one with a thick top coat (TC > 1 mm) and another with a thin layer (TC < 250 μm), selected to represent the extreme thickness range commonly reported for aerospace applications. By combining microstructural (SEM), surface analysis (XRD and roughness), and local mechanical testing (instrumented indentation and cross-sectional scratch testing, following the methodology proposed by Lopez et al. [13]) with high-temperature creep experiments at 125 MPa and 500 °C, we sought to establish correlations between coating microstructure, mechanical integrity, and creep behavior. Unlike previous studies that address these factors separately, we provide an integrated approach linking APS processing conditions, powder morphology, and coating thickness to damage evolution and creep behavior under conditions close to the operational limits of Ti-6Al-4V alloys. Post-creep coating integrity and fracture were analyzed using SEM and stereomicroscopy.

## 2. Materials and Methods

Two types of samples were prepared: cylindrical specimens with a thickness of 5 mm and specimens for creep testing, machined to the specific dimensions required by the available testing system, in accordance with ASTM E139 [14]. The Ti-6Al-4V alloy was used in the as-received condition, without any additional heat treatment prior to coating deposition, exhibiting a typical equiaxed α + β microstructure.

TBCs were applied by atmospheric plasma spraying (APS) onto Ti-6Al-4V substrates. Coating thicknesses were defined to represent the lower and upper bounds typically reported for TBC systems. Two coating systems were produced:

- TBC-1: NiCrAlY bond coat (Amperit 413.001, Höganäs, Scania County, Sweden) and ZrO_2_–8 wt% Y_2_O_3_ top coat (Amperit 831.054, Höganäs), deposited using a Praxair Miller Thermal SG-100 APS system with a Miller Thermal 1270 powder feeder.
- TBC-2: NiCrAlY bond coat (Amdry 962, Oerlikon Metco, Seoul, Republic of Korea) and ZrO_2_–8 wt% Y_2_O_3_ top coat (Metco 204NS, Oerlikon Metco), deposited using a ValuPlaz LCP Plasma Control Unit (Sulzer Metco, Brea, CA, USA).

Table 1 presents the APS parameters for the bond coat and top coat layers of TBC-1 and TBC-2.

The uniformity of the coatings was controlled during the APS deposition process by means of in-process monitoring of thickness and surface characteristics. Coating thickness was measured at multiple regions between deposition passes using a micrometer; surface characteristics were controlled using standard industrial roughness monitoring procedures. Despite a manually performed spraying procedure, these measures contributed to a consistent and reproducible coating structure.

The powders used in this study were commercially supplied materials with controlled particle size distributions provided by the manufacturer. Particle size distribution and powder morphology were analyzed by laser diffraction (CILAS 1190, CILAS, Orléans, France) and scanning electron microscopy (SEM, Tescan Vega3, Tescan, Brno, Czech Republic), respectively.

Phase analysis of the initial 8YSZ powder and the surface of the coated samples, providing information exclusively from the top coat layer, was performed using X-ray diffraction (XRD, Rigaku Ultima IV, Ann Arbor, MI, USA) with Cu Ka radiation (*λ* = 1.5418 Å), a scanning speed of 10° min^−1^, a step size of 0.02°, and operating conditions of 40 kV and 30 mA. A semi-quantitative analysis of lattice strain was performed based on XRD data. Interplanar spacing (*d*) was calculated using Bragg’s law [15], according to Equation (1):

(1) nλ=2dsinθ

in which *n* is the order of diffraction (usually *n* = 1), *λ* is the X-ray wavelength and *d* is the spacing between planes of given Miller indices *h*, *k*, and *l*. Additionally, the lattice strain (*ε*) was determined from the relative variation in interplanar spacing, an approach commonly used in XRD-based strain analysis [16]. Strain was calculated with respect to powder condition, considered as a stress-free reference, according to Equation (2):

(2) $$\varepsilon =\frac{\left(d-d_{0}\right)}{d_{0}}$$

in which *d*_0_ corresponds to the powder condition, assumed as a stress-free reference. Only well-defined and non-overlapping diffraction peaks of the tetragonal phase were selected for analysis. Full width at half maximum (FWHM) of the selected diffraction peaks was obtained by individual peak fitting, and values were expressed in degrees (2*θ*). Peak broadening was further analyzed using the Williamson–Hall method [17] to evaluate microstrain contributions.

Scanning electron microscopy (SEM, VEGA3 and MIRA3, TESCAN, Brno, Czech Republic) characterized cross-sectional regions of the sprayed coatings. Metallographic preparation was conducted using a semiautomatic polisher (Tegramin-20, Struers, Ballerup, Denmark) involving grinding with MD-MEZZO 220, fine grinding with MD-LARGO and a 9 mm diamond suspension, final polishing with colloidal silica (MD-CHEM), and subsequently etching with Kroll’s reagent (5 mL HNO_3_, 3 mL HF, and 100 mL of distilled water). Porosity was quantified from SEM cross-sectional images using ImageJ software (version 1.4.3.x). The obtained images were converted into binary format, and the porosity fraction was calculated as the ratio between pore area and total analyzed area. At least two representative regions were analyzed for each coating condition.

Surface roughness was measured using a profilometer (Mitutoyo SJ-210, Kawasaki, Japan), following ISO 4287-1997 [18] using a Gaussian filter. Roughness parameters *Ra*, *Rq*, and *Rz* were obtained as the average of five measurements per coating. Indentation hardness (HIT) and indentation elastic modulus (EIT) of the coatings were evaluated before and after creep testing by instrumented indentation using an Anton Paar system equipped with a Vickers indenter (V-J09, Anton Paar GmbH, Graz, Austria). Data acquisition was performed at a frequency of 10 Hz, using a Dz displacement sensor to ensure high measurement accuracy. Scratch tests were performed on the cross-section using an Anton Paar scratch tester equipped with a Rockwell diamond indenter (100 µm radius), under a constant normal load of 3 N. Tests were conducted in triplicate. Due to the inherent heterogeneity of plasma-sprayed coatings, variations in damage evolution along the scratch track were observed. Representative and reproducible responses exhibiting consistent damage progression were considered for quantitative analysis. Following the methodology proposed by Lopez et al., damage geometry was quantified by the longitudinal (*L_y_*) and transverse (*L_x_*) damage lengths, the damage cone angle (*α*), and the projected cone area [13].

Constant-load creep tests were performed on air using a standard creep testing machine (Kappa DS, Zwick/Roell, Ulm, Germany) equipped with a three-zone furnace and a computer-based control and data acquisition system (TestXpert II software, version 3.71), in accordance with ASTM E139 [14]. All tests were conducted at 500 °C under a constant stress of 125 MPa. Three specimen conditions were evaluated: uncoated Ti-6Al-4V, Ti-6Al-4V coated with TBC-1, and Ti-6Al-4V coated with TBC-2. A single test was performed per condition, with results showing consistent creep behavior. Fractographic analysis after creep testing was performed using SEM (SEM, VEGA3, TESCAN) and optical stereomicroscopy (SZ61, Olympus, Tokyo, Japan) equipped with an auxiliary lens (110AL2X, Olympus, Tokyo, Japan), with image analysis performed using LCMicro software (version 2.4, Build 29191).

## 3. Results and Discussion

### 3.1. Initial Powder Characterization

**Figure 2:**
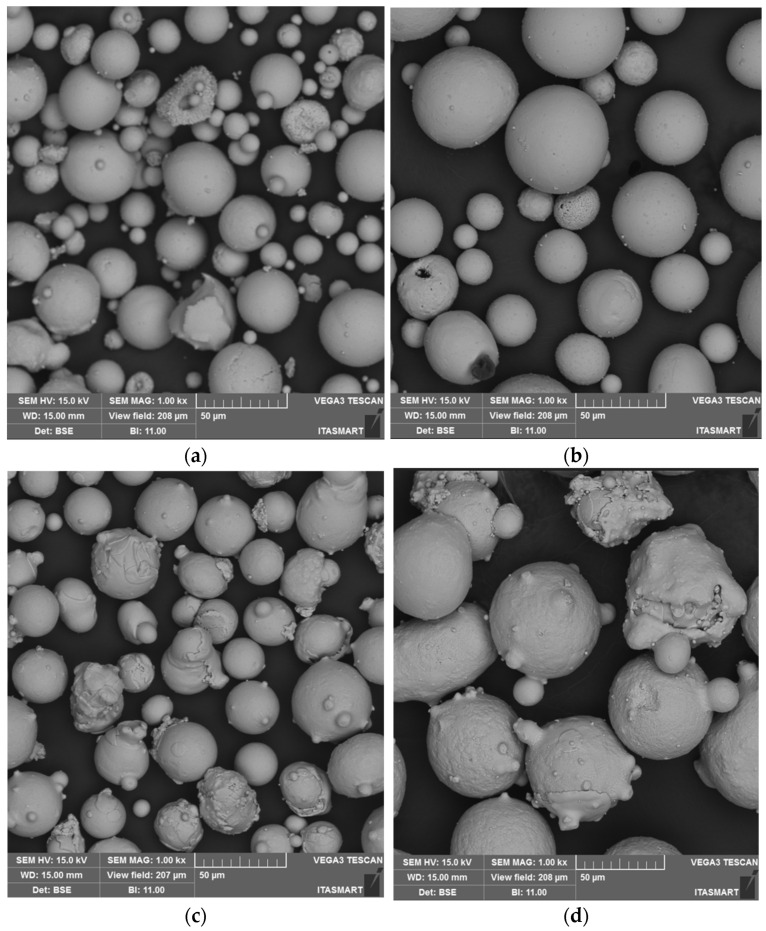
SEM micrographs of the 8YSZ and NiCrAlY powders used for TBC-1 (**a**,**c**) and TBC-2 (**b**,**d**), showing differences in particle morphology and size distribution.

The ceramic and metallic powders used for TBC-1 and TBC-2 were first characterized by scanning electron microscopy (SEM). Figure 2 shows SEM micrographs of the 8YSZ and NiCrAlY powders used in the TBC-1 and TBC-2 systems. The 8YSZ powder for TBC-2 (Figure 2b) presents a predominantly spherical morphology with a broad particle size distribution, typical of agglomerated and sintered ceramic powders for APS. The particles exhibit smooth surfaces with occasional porosity, consistent with micron-sized spherical powders reported in the literature [19], which promote stable powder feeding and excellent flowability during spraying [2]. Similarly, the NiCrAlY powder used for TBC-2 (Figure 2d) presents a predominantly spherical morphology and uniform particle sizes with minimal morphological irregularities, indicating good compositional consistency [5].

Conversely, the 8YSZ powder employed for TBC-1 (Figure 2a) presents more pronounced surface irregularities, with some particles showing hollow or partially dense structures [6]. Also notable is a broader range of particle sizes compared with the TBC-2 powder. The NiCrAlY powder used for TBC-1 (Figure 2c) shows similar differences in particle size distribution. These differences were later quantitatively confirmed by particle size distribution analysis.

**Figure 3:**
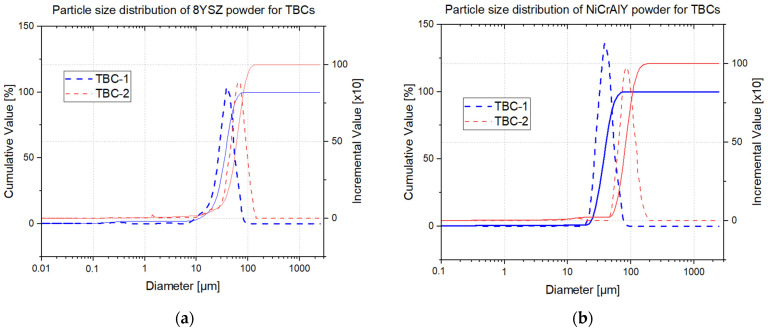
Particle size distributions of the initial powders used for TBC-1 and TBC-2: (**a**) 8YSZ top coat powders and (**b**) NiCrAlY bond coat powders. Solid lines represent cumulative particle size distributions, while dashed lines represent incremental particle size distributions.

Table 2 summarizes the particle size distribution parameters obtained for the ceramic and metallic powders. Figure 3 shows the cumulative and incremental particle size distributions for the 8YSZ and NiCrAlY powders.

The ceramic powders employed for both systems exhibit broad size distributions, with notable differences. The 8YSZ powder used for TBC-2 presents larger characteristic particle sizes, with D_10_, D_50_, and D_90_ values of 33.01, 60.40, and 91.31 μm, respectively, compared with 18.27, 35.33, and 53.86 μm for the TBC-1 powder. This corresponds to increases of 81%, 71%, and 70%, respectively. These results confirm the qualitative SEM analysis observations, which indicated a wider size range and a higher fraction of coarse particles for the TBC-2 ceramic powder. Similar trend occurred for the NiCrAlY bond coat powders, with the TBC-2 powder presenting larger particle sizes (D_50_ = 83.44 μm) about 123% larger than that of TBC-1 (D_50_ = 37.37 μm) along with a broader overall distribution.

The shift in the cumulative distribution curves toward larger diameters for TBC-2 (Figure 3b) indicates a higher proportion of coarse particles. These differences are critical for understanding the microstructure of the final coatings, as smaller powder particles are generally reported to melt more completely during spraying, resulting in denser coatings with lower porosity [7].

### 3.2. Coatings Microstructures and Thicknesses

**Figure 4:**
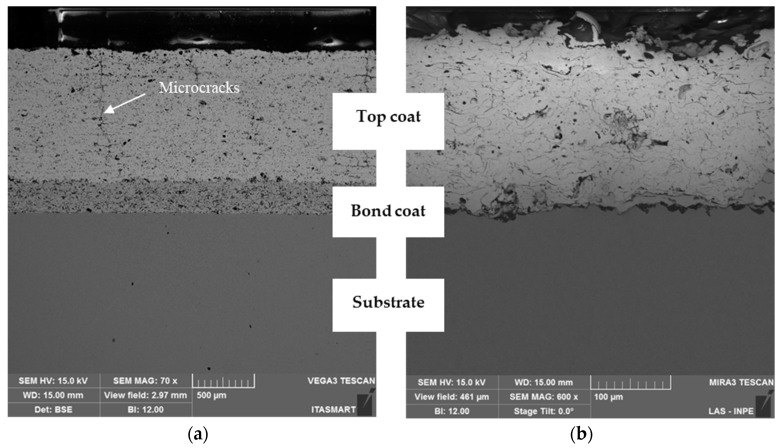
Cross-sectional SEM micrographs of APS TBCs deposited on Ti-6Al-4V substrates: (**a**) TBC-1, showing segmented cracks; (**b**) TBC-2, crack-free. Different magnifications were used to enable visualization of the full coating systems due to the significant difference in layer thickness (TBC-1: thick; TBC-2: thin). Quantitative thickness values are provided in Table 3.

Figure 4 shows SEM cross-sectional micrographs of the thermal barrier coatings, illustrating the microstructure of the as-sprayed APS coatings deposited with powders of different sizes. The different magnifications used enabled visualization of the entire coating systems, given the significant differences in layer thickness between TBC-1 and TBC-2. Table 3 summarizes the top and bond coat thicknesses measured by SEM. TBC-1 presented a bond coat thickness of 275 ± 28 μm and a thick top coat of 1063 ± 35 μm, whereas TBC-2 featured a much thinner bond coat of 27 ± 6 μm and top coat of 202 ± 16 μm. Based on these measurements, TBC-1 can be classified as a thick TBC (TTBC) and TBC-2 as a thin TBC.

Despite the intentional increase in thickness, TBC-1 presented microcracks were observed, whereas TBC-2 remained crack-free (Figure 4a,b). The finer, hollow morphology of the TBC-1 powder likely reduced particle coalescence, promoting internal stresses during deposition [7]. Cracks form due to contraction stresses from solidifying melted particles, creating pores at vacant sites between unmelted or semi-molten particles [20,21]. In turn, the coarser TBC-2 particles promote better particle packing and densification, reducing stress accumulation and crack formation during spraying.

To quantify the microstructural differences observed in the SEM analysis, we evaluated the porosity of the coatings using image analysis (Table 4). Results indicate an average porosity of (4.70 ± 1.12)% for TBC-1 and (2.06 ± 0.58)% for TBC-2, corresponding to an increase of approximately 128% for TBC-1. The higher porosity observed in TBC-1 is associated with the finer and partially hollow powder morphology, which can lead to incomplete densification during splat formation and promote the formation of interlamellar voids. Although smaller particles are generally reported to enhance melting efficiency and produce denser coatings [7], hollow structures present in TBC-1 modify this behavior by reducing effective particle cohesion during deposition. In contrast, the coarser and denser particles in TBC-2 promote improved particle packing and splat cohesion during impact and solidification, resulting in a more compact microstructure with lower porosity [22]. These results underscore that powder characteristics, particularly particle size distribution and morphology, are key factors in defining coating microstructure and integrity, alongside conventional APS parameters.

The combined effect of increased top coat thickness and high porosity in TBC-1 also contribute to higher thermal stress during deposition. Larger temperature gradients reduce thermal shock resistance and bond strength, promoting a mismatch in expansion or contraction within the coating. This thermal stress mismatch can lead to premature spalling or failure under cyclic hot and cold service conditions [23]. Segmented cracks, as observed in TBC-1, may help relieve stresses from TGO growth, improving strain tolerance and potentially extending coating service life [24,25,26,27]. For atmospheric plasma-sprayed coatings, thicker ceramic layers induce larger temperature gradients, causing splat shrinkage and the formation of microcracks which can connect and form segmented cracks through the coating thickness.

### 3.3. Surface Characteristics

Table 5 summarizes the surface roughness measurements of the top coats for TBC-1 and TBC-2, and the uncoated Ti-6Al-4V substrate. Both coated systems present significantly higher roughness values compared with the uncoated substrate, as expected for plasma-sprayed ceramic coatings.

We observed a clear difference in surface roughness between TBC-1 and TBC-2. TBC-2 shows higher *Ra*, *Rq*, and *Rz* values, indicating a rougher and more irregular surface morphology. Quantitatively, *Ra* and *Rq* values for TBC-2 are about 70–75% higher than those for TBC-1, whereas Rz increases by approximately 60%, underscoring the strong influence of processing parameters on surface roughness. This behavior is attributed to both powder characteristics and coating thickness. The smaller particle size of TBC-1 powder leads to thinner, more homogeneous lamellae, resulting in a smoother surface [6,19]. In turn, the coarser particles used for TBC-2 retain more pronounced surface asperities.

Coating thickness also plays an important role in roughness. As noted, TBC-1 is a thick TBC (TTBC) and TBC-2 a thin TBC. Thicker coatings tend to undergo repeated splat stacking, leading to partial surface smoothing as successive layers are deposited. Conversely, thinner coatings preserve surface features associated with individual splats, resulting in higher roughness. The elevated *Rq* and *Rz* values for TBC-2 indicate more pronounced peaks and valleys, which may influence stress concentration and thermal fatigue behavior during service [28]. While increased roughness can enhance mechanical interlocking and adhesion, excessive surface irregularity may increase thermal stresses, potentially compromising coating durability under cyclic thermal loading [29].

### 3.4. Integrity of the Coatings

Hardness and elastic modulus are key properties for TBC performance, as they are strongly influenced by microstructure, porosity, and particle characteristics of the coatings [30,31]. Table 6 summarizes the significant differences observed between the top coats of the samples. TBC-1 exhibited slightly lower hardness (approximately 5% lower) but a significantly higher elastic modulus (approximately 58% higher) than TBC-2. These results suggest that the TBC-1 top coat is mechanically stiffer and more resistant to penetration, whereas TBC-2 is more compliant, favoring elastic and inelastic deformation under applied loads [32,33]. Comparatively, the substrate showed similar hardness and modulus values for both systems, confirming that the mechanical differences are primarily due to the coating architecture, not the metallic substrate.

These differences are attributed to the characteristics of the 8YSZ powders and APS conditions. The smaller and more widely size-distributed 8YSZ powder used for TBC-1 likely melted more completely during spraying, producing locally denser lamellae and stronger inter-lamellar bonding [33]. This local densification at the splat level increases hardness and elastic modulus by minimizing local deformation at pores or micro-defects. However, TBC-1 presented higher overall porosity and also contained pre-existing cracks (Figure 4a), which may act as stress concentrators under loading, partially offsetting the benefits of higher stiffness [5]. Conversely, the coarser 8YSZ powder used for TBC-2 likely melted incompletely, leading to weaker lamellar bonding. This results in a more compliant top coat, which accommodates both elastic and inelastic deformation, facilitating stress redistribution within the multilayer system [34].

**Figure 5:**
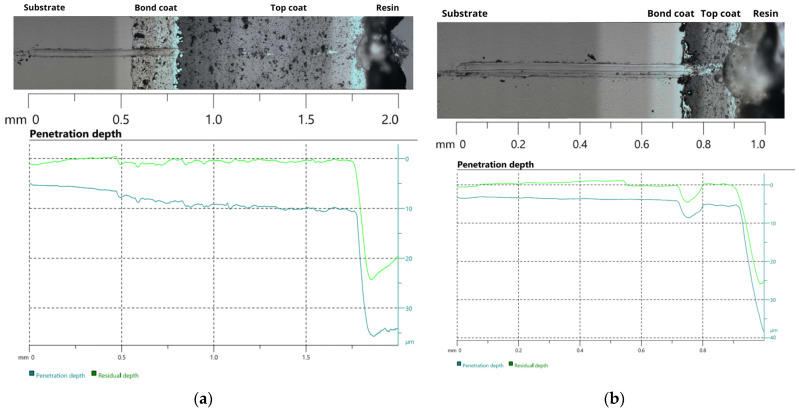
Penetration and residual depth profiles and corresponding cross-sectional images from scratch tests of (**a**) TBC-1 and (**b**) TBC-2.

Scratch test results confirmed distinct mechanical responses between the TBC-1 and TBC-2 systems, particularly in the evolution of penetration depth along the scratch path (Figure 5). Although both coatings were tested under identical loading conditions and exhibited penetration depths of the same order of magnitude, TBC-2 showed more pronounced variations in both penetration and residual depth upon approaching the bond coat region (Figure 5b). This behavior is consistent with localized damage events, including interlamellar cracking and porosity collapse, often observed in thermally sprayed ceramic coatings and intensifying near interfaces with strong mechanical contrast [35]. Conversely, TBC-1 displayed a more stable penetration profile throughout the top coat, followed by a smoother transition at the top coat/bond coat interface (Figure 5a), suggesting better load accommodation and higher resistance to localized deformation within the coating.

Despite these differences in penetration behavior, the scratch damage geometry parameters (L_x_, L_y_, α, and projected cone area [13]) were similar for both coatings (Table 7).

This indicates that final scratch geometry alone is not sufficient to distinguish the underlying damage mechanisms. Similar observations have been reported in tribomechanical studies of brittle coatings, where comparable scratch geometries were obtained despite significant differences in adhesion, deformation mechanisms, and damage accumulation during scratching [36]. Thus, the evolution of penetration and residual depth provides a more meaningful assessment of the mechanical integrity of APS TBC systems than post-test geometric parameters alone.

### 3.5. High-Temperature Creep Behavior

**Figure 6:**
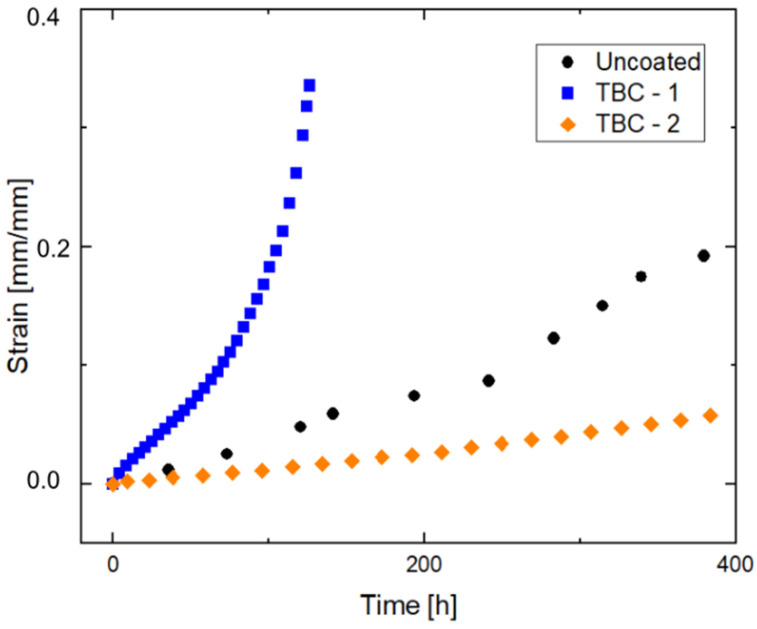
Creep curves of the Ti-6Al-4V alloy at 500 °C and 125 MPa for the following conditions: uncoated sample, coated sample TBC-1 and coated sample TBC-2, emphasizing the interval up to 400 h.

Figure 6 presents the creep curves of strain (ε) versus time (h) for TBC-1, TBC-2, and the uncoated Ti-6Al-4V substrate at 500 °C under an applied stress of 125 MPa. For comparison purposes, the figure highlights the creep behavior within the first 400 h of testing.

Table 8 summarizes the main parameters obtained from the creep tests. Here, *t_p_* corresponds to the primary creep stage time, *ε̇_s_* denotes the steady-state creep rate, *t_r_* represents the time to rupture, *ε_f_* is the strain at fracture, and RA refers to the reduction in area after failure.

Creep behavior in Ti-6Al-4V is known to strongly depend on phase morphology and grain structure [37]. Reported steady-state creep rates for this alloy vary significantly depending on processing route and testing conditions, typically ranging from 10^−8^ to 10^−6^ s^−1^ under stresses between 250 and 350 MPa at 500 °C [9]. This variability reflects the strong influence of microstructural features on deformation mechanisms, as recent studies have demonstrated that creep in this alloy, in the uncoated condition, is governed by a combination of dislocation glide and climb, controlled by phase distribution and lamellar morphology [9]. In the present study, the steady-state creep rate obtained for the uncoated substrate (0.0006 h^−1^, equivalent to approximately 1.7 × 10^−7^ s^−1^) falls within this range, despite the lower applied stress (125 MPa), indicating consistency with the literature and reinforcing the important role of microstructural factors in controlling creep rate. In addition, here the creep response of the Ti-6Al-4V alloy was strongly influenced by the presence and thickness of the thermal barrier coatings. Particularly relevant among the measured parameters is the steady-state creep rate (*ε̇_s_*), as it reflects the resistance of the material to plastic deformation under the applied temperature and stress, with higher *ε̇_s_* and lower *t_r_* values indicating lower creep resistance [38]. To better illustrate this, *t_r_* and *ε̇_s_* for the coated samples were normalized to the uncoated substrate (Table 9).

As shown in Table 8, the uncoated substrate exhibited a relatively low steady-state creep rate (0.0006 h^−1^) and a long time to rupture (904 h), which can be consistent with a predominantly ductile creep response. Conversely, the thicker coating (TBC-1) presented a significantly higher *ε̇_s_* and a substantial reduction in *t_r_* (Table 8), indicating a detrimental effect of increased coating thickness on creep resistance under the investigated conditions. This behavior is associated with the finer and partially hollow powder morphology of TBC-1, and segmented microcracks present within the top coat (Figure 4a) which act as preferential sites for crack initiation and propagation, accelerating damage accumulation and reducing creep life [39]. Additionally, it is also influenced by the higher porosity observed in TBC-1, which reduces load-bearing capacity and promotes stress concentration, facilitating crack initiation and accelerating damage accumulation during creep [7,20,21].

However, the deterioration in creep performance cannot be attributed solely to these microstructural defects. In thicker coatings, higher thermal gradients during deposition and cooling are expected to promote the development of residual stresses within the ceramic layer, as widely reported for plasma-sprayed TBC systems [40,41,42]. These thermal gradients, combined with the heterogeneous and lamellar nature of APS coatings, may lead to complex heat transfer behavior influenced by porosity, interfaces, and microcracks. Although alternative modeling approaches, such as non-classical heat conduction models, have been proposed for complex materials [43], such effects were not directly investigated in the present study. Nevertheless, the resulting residual stresses are expected to increase the effective stress acting on the coating/substrate system during creep, promoting localized deformation and accelerating damage accumulation. Moreover, the increased thickness of TBC-1 enhances stress gradients across the coating thickness, which may lead to strain incompatibility between adjacent lamellae and between coating layers. Such effects can intensify stress concentration, particularly under sustained loading, further promoting crack propagation [3,5] and reducing creep lifetime. Conversely, the thinner coating (TBC-2) showed a lower *ε̇_s_* and a moderately reduced *t_r_*, indicating improved creep resistance relative to TBC-1 despite a shorter absolute lifetime compared with the uncoated alloy. Nonetheless, the combination of low *ε̇_s_*, high accumulated strain, and a substantial reduction in area (77.92%) suggests delayed damage evolution rather than premature failure. These results highlight the interdependence between microstructure, mechanical response, and creep behavior.

To further elucidate the role of coating thickness on creep behavior, we compared our results with selected literature data obtained under similar testing conditions (500 °C and 125 MPa). De Freitas et al. [44] investigated a TBC system using the same 8YSZ and NiCrAlY powders as TBC-2, with bond coat and top coat thicknesses of approximately 67 and 284 μm, respectively. They reported a steady-state creep rate of 0.0002 h^−1^ and a time to rupture of approximately 409 h. Comparetively, TBC-2 in the present study exhibited a similar *ε̇_s_* (0.0002 h^−1^), but a longer time to rupture (576 h). The high accumulated strain and a reduction in area of 78% observed for TBC-2 suggest that the thinner coating favored stress redistribution during creep, delaying catastrophic failure.

Other direct quantitative comparisons with literature data are limited due to differences in powder characteristics and coating thickness. However, studies employing thicker and optimized TBC have reported lower steady-state creep rates and longer rupture times. Briguente et al. [45] investigated the creep behavior of uncoated, bond-coated, and TBC-coated Ti-6Al-4V under comparable conditions (500 °C and 125 MPa). They reported that both bond coat and TBC systems significantly improved creep resistance relative to the uncoated alloy, resulting in reduced *ε̇_s_* and extended rupture lifetimes, up to 131% higher than uncoated results. Together, these findings, along with the present study, highlight the critical role of coating design in controlling creep performance.

Within this context, scratch test results provide complementary insight into the mechanical response of the coating systems, but do not directly predict creep behavior. TBC-2 exhibited a higher penetration depth and greater deformation accumulation within the coating system, particularly at the bond coat region. In turn, TBC-1 showed a more restricted penetration profile, suggesting a stiffer and more constrained system, which promotes higher stress retention within the coating during mechanical loading [36]. Under sustained creep conditions, such stress retention in TBC-1 may accelerate damage accumulation and crack propagation [35], contributing to the higher steady-state creep rate and reduced rupture time observed for TBC-1.

### 3.6. Fracture and Damage Analysis of Crept Samples

**Figure 7:**
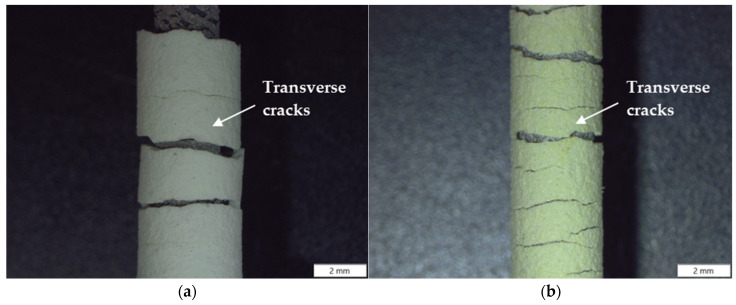
Stereoscopic images of crept samples: (**a**) TBC-1 and (**b**) TBC-2, highlighting the presence of transverse cracks perpendicular to the applied stress direction.

Fracture and damage mechanisms in the crept samples were first assessed by macroscopic stereomicroscopy of the fracture surfaces. Figure 7 presents stereoscopic images of TBC-1 and TBC-2 samples after creep testing conducted at 125 MPa and 500 °C.

Both coated systems exhibit transverse cracking in the ceramic top coat, aligned perpendicular to the applied stress, indicating tensile stress accumulation during creep. However, we observed a significant difference in cracking behavior between the thick and thin TBC systems. The thick TBC sample (Figure 7a) shows fewer but more pronounced cracks, indicative of rapid crack propagation and localized damage accumulation. This behavior is consistent with higher residual stresses typically associated with thicker plasma-sprayed ceramic layers [7], compounded by the presence of pre-existing microcracks observed in the as-deposited condition (Figure 4a). Such defects are known to act as preferential crack propagation paths, accelerating coating degradation and promoting delamination [46].

Here, the higher secondary gas flow rate used for TBC-1 deposition (H_2_ = 21 L min^−1^, Table 1) likely increased particle velocity and splat quenching rates, promoting higher residual stresses and a more constrained ceramic layer [34,47]. Under combined thermal exposure and sustained mechanical loading, these features favor stress localization and rapid damage progression [3,24,25]. Additionally, microstructural heterogeneities inherent to APS coatings, such as pores, voids, and partially unmelted regions, serve as local stress concentrators under tensile and creep deformation, further facilitating crack initiation and growth [48]. As coating integrity progressively deteriorates, its thermal and protective effectiveness diminishes, increasing substrate exposure to oxidation and thermomechanical stresses. The elastic modulus and fracture toughness of the ceramic layer therefore play a critical role in governing crack driving forces, as discussed by Gao et al. [34]. These factors collectively contribute to the reduced creep time to fracture and to the higher secondary creep rate observed for the TBC-1 system.

Comparatively, the thin TBC sample (Figure 7b) exhibits a higher density of distributed transverse cracks along the top coat. Rather than indicating premature failure, this cracking pattern suggests an effective stress-relief mechanism, enabling partial relaxation of thermally and mechanically induced stresses during creep exposure. Yang et al. [3] evinced that intentionally segmented surface cracks in the plasma-sprayed top coat can function as effective stress-relief features, enhancing strain tolerance and improving service durability. Although not intentionally engineered here, the formation of multiple transverse cracks in TBC-2 appears to play a similar role, contributing to improved creep resistance when compared with TBC-1. This behavior supports the notion that, under creep conditions, a controlled crack density may be less detrimental than a highly constrained coating subjected to elevated residual stresses.

An important aspect highlighted by the combined scratch and creep analyses is the role of the bond coat in influencing damage evolution within the coating system. Scratch tests revealed that TBC-2 experienced a pronounced increase in penetration depth upon reaching the bond coat, indicating that this layer participated actively in deformation accommodation. Under creep conditions, such behavior is consistent with a more gradual transfer of stresses from the ceramic top coat to the metallic substrate, reducing interfacial stress concentration. Conversely, the bond coat in TBC-1 exhibited a more constrained mechanical response during scratch testing, suggesting limited stress accommodation. During sustained creep exposure, this condition likely promotes stress retention at the interface, accelerating damage accumulation and contributing to the reduced creep lifetime of this system.

**Figure 8:**
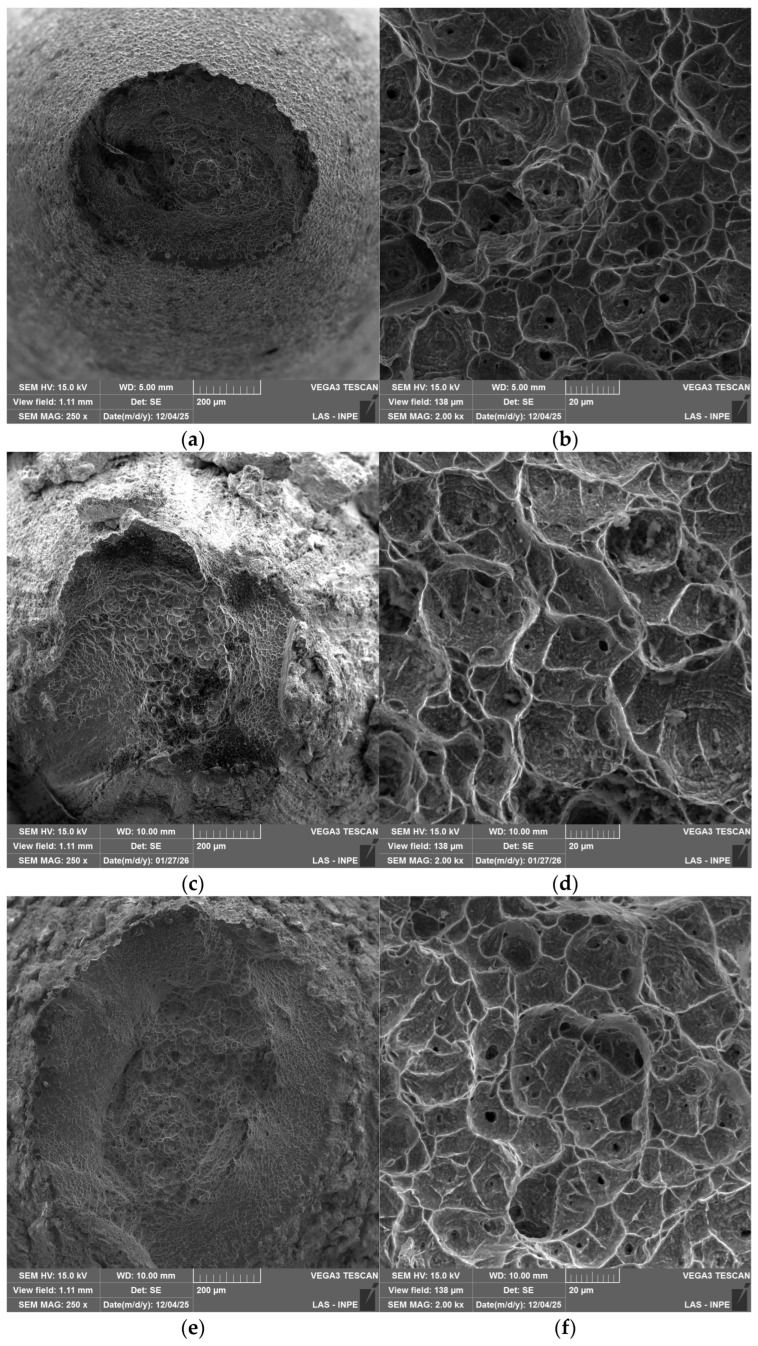
Fracture morphology by SEM after creep test at 125 MPa and 500 °C: (**a**,**b**) uncoated sample; (**c**,**d**) TBC-1; and (**e**,**f**) TBC-2.

SEM examination of the fracture surfaces (Figure 8) revealed similar fracture morphologies for uncoated and coated samples. All conditions presented a cup-and-cone morphology with a dimpled appearance, characteristic of ductile failure. This observation is consistent with the high reduction in area values measured for all samples (Table 7), indicating that coating application did not alter the dominant ductile fracture mechanism of the Ti-6Al-4V substrate. Additionally, a shear zone with an inclination of approximately 45° relative to the loading axis occurred near the specimen edges, which is typical of ductile fracture under tensile loading [44]. Higher magnification images reveal the presence of equiaxed dimples, further confirming microvoid nucleation, growth, and coalescence as the ruling fracture process. Despite differences in creep lifetime and steady-state creep rate, fracture remained predominantly ductile, indicating that coating-related effects primarily influenced damage kinetics rather than the fundamental failure mode of the substrate.

### 3.7. Residual Stress Evolution and Mechanical Response After Creep

**Figure 9:**
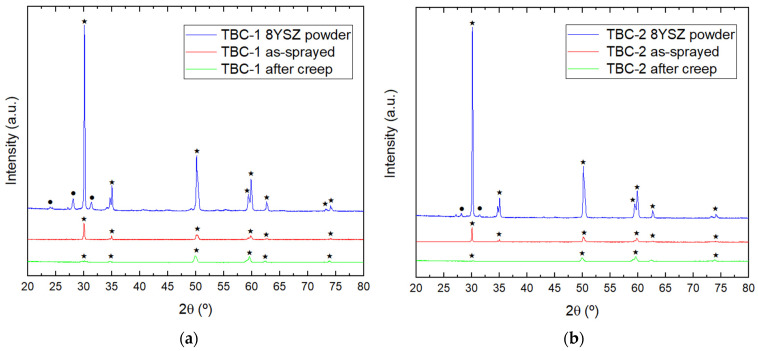
XRD of YSZ powders and as-sprayed coatings of (**a**) TBC-1 and (**b**) TBC-2, where ★ represents tetragonal metastable phase (t′-ZrO_2_) and ● indicates monoclinic phase (m-ZrO_2_).

XRD analysis investigated phase stability, lattice strain, and microstructural evolution of the coatings before and after creep exposure. Figure 9 shows the diffraction patterns of the YSZ powders, as-sprayed coatings (TBC-1 and TBC-2) and crept samples. All patterns exhibit the tetragonal phase (t′-ZrO_2_), which remains metastable at room temperature due to yttria doping. The Y_2_O_3_ present in 8YSZ prevents its transformation to monoclinic (m-ZrO_2_) after the high-temperature deposition process and fast cooling [6,49]. Phase identification used ICSD reference patterns and HighScore Plus software (version 2.0a), with the tetragonal phase indexed according to ICSD code 075309 (Zr_0.9_Y_0.1_O_1.95_, PDF 01-082-1241) and the monoclinic phase according to ICSD code 080048 (PDF 01-083-0942).

Regarding microstructure, all powders show t′-ZrO_2_ as the dominant phase, with a small fraction of m-ZrO_2_. In contrast, XRD resolution detected no monoclinic phase in the as-sprayed coatings, indicating a successfully inhibited phase transition during the spraying process. This inhibition is critical since phase transformations between the t’-ZrO_2_ and m-ZrO_2_ phases involve significant volume changes. When zirconia cools from high temperatures, the tetragonal to monoclinic phase transformation is martensitic and results in a volume increase of 3–5% [1]. Such changes induce internal stresses, which could lead to coating delamination and failure, particularly under thermal cycling conditions. As discussed, after performing creep tests, the samples exhibited cracking and spallation. However, XRD analysis of the crept samples revealed no phase transformation, confirming that the observed failure was not related to the t′-ZrO_2_ to m-ZrO_2_ transformation. This suggests that the failure mechanisms, including cracking and spallation, were likely due to other factors, rather than phase transformation.

A semi-quantitative lattice strain (*ε*) analysis using selected tetragonal diffraction peaks further investigated residual stress evolution. Table 10 summarizes the lattice strain values for TBC-1 and TBC-2 before (as-sprayed) and after creep exposure calculated according to Equation (2). Results reveal a heterogeneous strain state, with both positive and negative ε values depending on the diffraction plane. This indicates that residual stresses are not uniformly distributed within the coatings, reflecting the intrinsic anisotropy and heterogeneity of plasma-sprayed microstructures.

After creep exposure, we observed no systematic trend in Δ*ε*, which indicates a complex redistribution of residual stresses. Negative Δ*ε* values suggest local stress relaxation or the development of compressive stresses, whereas positive Δ*ε* values are associated with lattice distortion induced by defect accumulation and microstructural damage during high-temperature exposure [40,41,42].

Heterogeneous strain evolution contributes to crack initiation and propagation, particularly under creep conditions, in which localized stress concentrations act as driving forces for damage accumulation. These mechanisms are consistent with the experimentally observed cracking patterns and spallation of the ceramic top coat, especially in the thicker coating (TBC-1).

In addition to lattice strain, peak broadening was evaluated by the full width at half maximum (FWHM) of selected diffraction peaks (Table 11). The FWHM values provide insight into microstrain and defect density within the coatings. Changes in FWHM after creep exposure indicate microstructural evolution, including the formation of microcracks, increased defect density, and redistribution of internal stresses.

**Figure 10:**
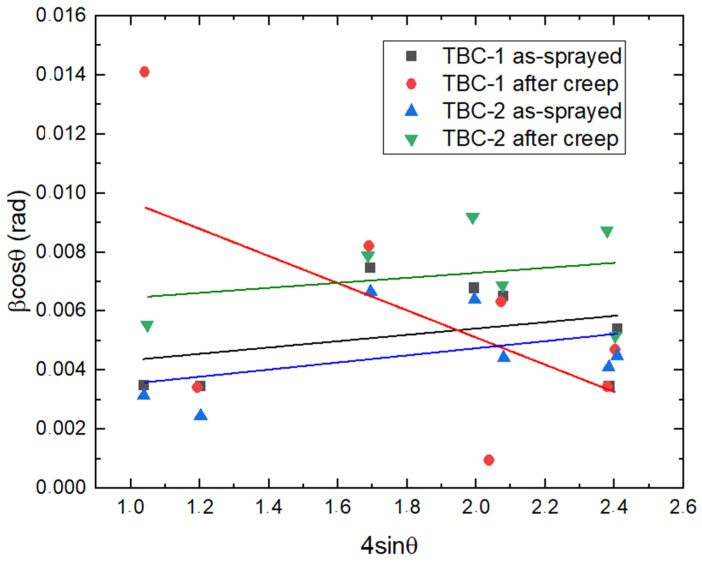
Williamson–Hall plots for TBC-1 and TBC-2 coatings in as-sprayed and after creep conditions. Linear fits were applied to each dataset. The dispersion of the data and deviations from linearity reflect the heterogeneous microstructure of plasma-sprayed coatings.

Williamson–Hall (W–H) analysis estimated microstrain from peak broadening, as shown in the corresponding plots in Figure 10. However, the results showed limited linear correlation and, in some cases, non-physical slopes. This behavior is attributed to the intrinsic heterogeneity, anisotropy, and porosity of plasma-sprayed coatings, which violate the assumptions of isotropic peak broadening required for ideal W–H analysis [17]. Consequently, direct evaluation of lattice strain (*ε*) and FWHM was considered more reliable for assessing microstructural distortion and residual stress evolution in these coatings in our study.

Comparatively, TBC-1 exhibits more pronounced variations in both lattice strain and peak broadening, suggesting a more constrained microstructure with higher internal stress accumulation. This condition is consistent with the higher effective stress and accelerated damage evolution observed during creep. Conversely, TBC-2 shows more moderate changes, indicating a greater ability to accommodate deformation and redistribute stresses, which is supported by the scratch test results that revealed higher penetration variability and localized deformation mechanisms, particularly near the bond coat region, indicative of a more compliant mechanical response. Such behavior is directly associated with the improved creep performance and reduced damage accumulation observed for the thinner coating, as well as porosity measurements where TBC-1 exhibited a higher porosity level. Presence of pores and interlamellar defects contributes to heterogeneous strain distribution and increased peak broadening, reinforcing the relation between microstructure and residual stress evolution. Similar relations between porosity, defect density, and residual stress evolution have been widely reported for plasma-sprayed coatings [20,21,40].

The evolution of residual stresses and their mechanical implications was investigated by instrumented indentation measurements performed after creep exposure (Table 12).

For TBC-1, the substrate exhibited a significant increase in hardness after creep, rising from 4441 MPa in the as-sprayed condition (Table 6) to 6394 MPa (Table 12), corresponding to an increase of approximately 44%. This was accompanied by a pronounced reduction in elastic modulus, from 147 GPa (Table 6) to 93 GPa (Table 12), indicating localized plastic deformation and strain hardening associated with stress accumulation and limited stress relaxation. Such behavior has been reported in metallic substrates subjected to high-temperature deformation, where dislocation interactions and defect accumulation lead to increased hardness and reduced elastic stiffness [9]. Conversely, the substrate under TBC-2 showed a slight decrease in hardness, from 5834 MPa in the as-sprayed condition (Table 6) to 5639 MPa (Table 12), along with a minor reduction in elastic modulus, suggesting the activation of recovery mechanisms and more effective stress redistribution during creep [11]. A similar trend is observed in the top coat, in which TBC-1 shows a more pronounced increase in hardness after creep, indicating a more constrained mechanical response, whereas TBC-2 exhibits more moderate changes. These results provide mechanical evidence of the different stress evolution mechanisms inferred from the XRD analysis.

The combined analysis of lattice strain, peak broadening, and post-creep indentation results indicates that TBC-1 promotes stress accumulation and localized deformation, whereas TBC-2 enables progressive stress redistribution and deformation accommodation. These findings are consistent with the creep behavior observed, since the reports of deformation in Ti-6Al-4V show that it is governed by dislocation glide and climb [9]. Thus, the ability of the coating system to redistribute or accumulate stresses directly influences the localization of deformation mechanisms, ultimately controlling creep resistance.

**Figure 11:**
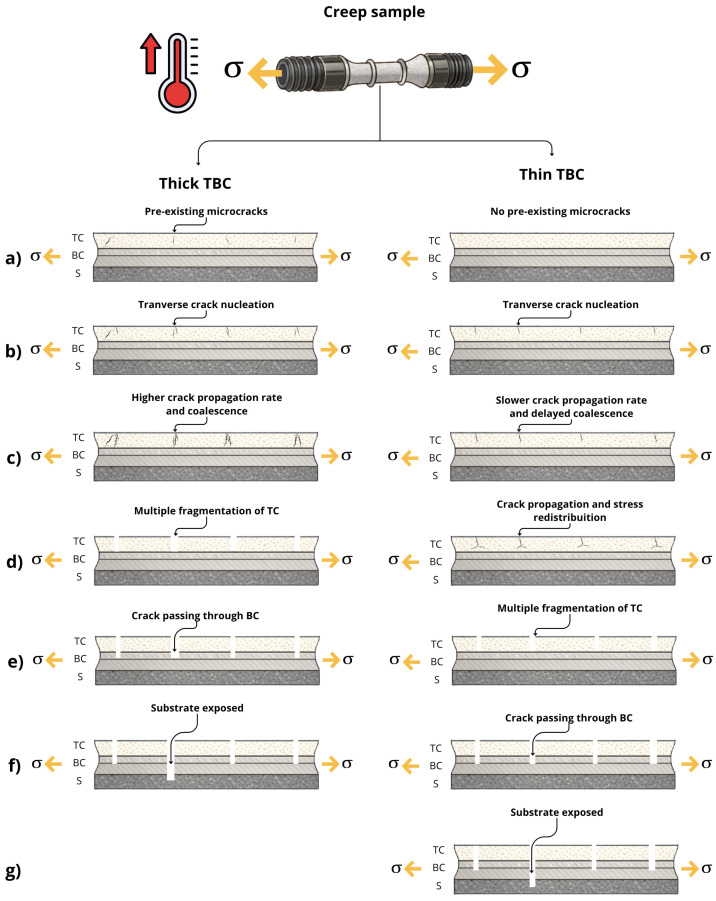
Schematic and qualitative representation of damage evolution in thick and thin plasma-sprayed thermal barrier coatings (TBCs) under creep conditions. Letters (**a**–**g**) indicate successive stages of damage development, from crack nucleation to coating failure and substrate exposure. The scheme highlights the contrasting cracking behavior of thick and thin TBCs and is intended as a conceptual interpretation rather than a quantitative description.

As a visual summary of these observations, Figure 11 provides a schematic representation of damage evolution under creep. In this scheme, thin coatings exhibit distributed cracking that delays crack coalescence and promotes stress redistribution, whereas thick coatings experience accelerated crack propagation driven by higher residual stresses and limited stress-relief pathways. The schematic is intended as a qualitative interpretation based on this study, considering coating thickness, processing-induced residual stresses, and microstructural heterogeneities. Similar schematic models have been proposed for coatings under tensile or high-temperature loading [50,51] and were used as references for the present interpretation.

## 4. Conclusions

The following concluding remarks can be drawn based on the comparative assessment of uncoated Ti-6Al-4V substrates and plasma-sprayed thermal barrier coatings deposited under different process parameters:

- Powder morphology and particle size distribution influenced coating density and crack formation, thereby impacting the mechanical properties of the top coats.
- Thicker coating (TBC-1) exhibited higher elastic modulus with more homogeneous scratch behavior; however, segmented microcracks formed during deposition due to elevated residual thermal stresses.
- Thinner coating (TBC-2) presented lower elastic modulus, higher surface roughness, and greater mechanical compliance, while remaining initially free of microcracks.
- Scratch testing revealed distinct deformation mechanisms: TBC-2 exhibited higher penetration variability associated with localized deformation and stress accommodation, whereas TBC-1 showed a more restricted mechanical response.
- Creep testing at 500 °C and 125 MPa revealed that process parameters play a decisive role in time-dependent deformation: TBC-1 accelerated creep deformation and reduced time to rupture, whereas TBC-2 promoted improved stress redistribution and exhibited a lower steady-state creep rate.
- Creep behavior is not governed by coating thickness alone. Instead, it results from the combined influence of porosity, crack density, residual stresses, and mechanical compliance, which together control damage evolution and creep kinetics.
- Post-creep damage analysis revealed transverse cracking in both coatings, evincing the influence of residual stresses on long-term integrity.
- Post-creep indentation results revealed contrasting substrate responses: TBC-1 showed significant hardening and reduction in elastic modulus, indicating stress accumulation and limited relaxation, whereas TBC-2 exhibited minor changes, consistent with more effective stress redistribution during creep.
- Residual stress semi-quantitative analysis, supported by XRD and peak broadening, indicated that TBC-1 developed a more constrained and heterogeneous stress state, while TBC-2 promoted a more distributed stress evolution.
- Fractographic analysis confirmed a ductile fracture mode of the Ti-6Al-4V substrate for all conditions, indicating that the coatings primarily influenced damage evolution and creep kinetics rather than the fundamental fracture mechanism.

## Figures and Tables

**Table 1:** APS process for NiCrAlY bond coats and 8YSZ top coats.

Spraying Parameters	Units	BC-1 (NiCrAlY)	BC-2 (NiCrAlY)	TC-1 (8YSZ)	TC-2 (8YSZ)
Nozzle diameter	mm	7.3	7.7	7.3	7.7
Spraying current	Ampeére	800	550	700	600
Voltage	Volts	40	75	44	70
Standoff distance	mm	75–100	125	75–100	75
Powder feed rate	g min^−1^	25	45–50	50	50
Primary gas, Ar	L min^−1^	48	47	25	38
Secondary gas, H_2_	L min^−1^	20	7	21	7
Carrier gas	L min^−1^	8	9	8	9

Note 1. APS coating was performed under atmospheric conditions. Note 2. No substrate preheating was applied prior to coating deposition.

**Table 2:** Particle size distribution parameters of the 8YSZ top coat and NiCrAlY bond coat powders used for TBC-1 and TBC-2.

Layer	Sample	Variation [μm]	D_m_ [μm]	D_10_ [μm]	D_50_ [μm]	D_90_ [μm]
Bond coat (NiCrAlY)	TBC-1	0.04–90	38.66	26.33	37.37	53.10
	TBC-2	0.2–180	85.72	58.64	83.44	119.61
Top coat (8YSZ)	TBC-1	0.04–90	35.76	18.27	35.33	53.86
	TBC-2	0.07–140	61.45	33.01	60.40	91.31

**Table 3:** Measured thicknesses of bond and top coats for TBC-1 and TBC-2.

Layer	TBC-1 [μm]	TBC-2 [μm]
Bond coat	275 ± 28	27 ± 6
Top coat	1063 ± 35	202 ± 16

**Table 4:** Porosity values of the coatings TBC-1 and TBC-2.

Sample	Porosity [%]
TBC-1	4.70 ± 1.12
TBC-2	2.06 ± 0.58

**Table 5:** Surface roughness measurements of the top coat for samples TBC-1 and TBC-2, and of the uncoated Ti-6Al-4V substrate.

Sample	Ra [μm]	Rq [μm]	Rz [μm]
TBC-1	4.66 ± 0.16	5.87 ± 0.11	26.72 ± 1.19
TBC-2	8.22 ± 0.55	10.08 ± 0.72	43.62 ± 2.63
Uncoated	0.80 ± 0.13	1.05 ± 0.14	6.59 ± 0.37

**Table 6:** Instrumented indentation properties of the TBC-1 and TBC-2 samples.

Sample	Region Analyzed	HIT [MPa]	EIT [GPa]
TBC-1	Top coat	8070 ± 1023	152 ± 13
	Substrate	4441 ± 459	147 ± 5
TBC-2	Top coat	8524 ± 1482	96 ± 7
	Substrate	5834 ± 748	93 ± 3

**Table 7:** Scratch damage geometry parameters for TBC-1 and TBC-2.

Sample	Lx [μm]	Ly [μm]	α [°]	Projected Cone Area [μm^2^]
TBC-1	57	211	63	12,027
TBC-2	62	230	64	14,260

**Table 8:** Comparative table of the experimental creep parameters for the Ti-6Al-4V alloy with and without coating at 500 °C and 125 MPa.

Sample	*t_p_* [h]	*ε̇_s_* [h^−1^]	*t_r_* [h]	*ε_f_* [mm/mm]	RA [%]
Uncoated	35.84	0.0006	904.48	1.6499	84.93
TBC-1	16.80	0.0016	126.22	0.3359	82.67
TBC-2	23.17	0.0002	575.63 *	0.0929 *	77.92

* Interrupted creep test.

**Table 9:** Relative comparison of time to rupture (*t_r_*) and steady-state creep rate (*ε̇_s_*) for coated samples normalized to the uncoated substrate.

Sample	*t_r_*/*t_r_* Uncoated	*ε̇_s_*/*ε̇_s_* Uncoated
TBC-1	0.14	2.67
TBC-2	0.64	0.33

**Table 10:** Lattice strain (*ε*) and strain variation (Δ*ε*) for TBC-1 and TBC-2 coatings, calculated from selected tetragonal diffraction peaks.

Phase	(hkl)	ε As-Spray (TBC-1)	ε After-Creep (TBC-1)	Δε (TBC-1)	ε As-Spray (TBC-2)	ε After-Creep (TBC-2)	Δε (TBC-2)
t’-ZrO_2_	(101)	0.00247	−0.00065	−0.00312	0.00260	−0.00643	−0.00903
	(110)	0.00230	0.00949	0.00719	0.00222	-	−0.00222
	(112)	0.00048	0.00374	0.00326	0.00112	0.00563	0.00451
	(211)	0.00081	−0.02012	−0.02093	0.00091	0.00365	0.00274
	(202)	0.00013	0.00345	0.00332	0.00072	0.00287	0.00216
	(004)	0.00032	0.00236	0.00203	0.00071	0.00283	0.00212
	(220)	−0.00002	0.00302	0.00303	0.00069	0.00325	0.00256

**Table 11:** Peak broadening (FWHM) obtained from XRD patterns for TBC-1 and TBC-2 coatings, indicating microstrain and defect evolution before and after creep exposure.

Phase	(hkl)	FWHM As-Sprayed (TBC-1) [° 2θ]	FWHM After Creep (TBC-1) [° 2θ]	FWHM As-Sprayed (TBC-2) [° 2θ]	FWHM After Creep (TBC-2) [° 2θ]
t’-ZrO_2_	(101)	0.2063	0.8367	0.1870	0.3288
	(110)	0.2071	0.2056	0.1475	-
	(112)	0.4717	0.5186	0.4211	0.4983
	(211)	0.4485	0.0630	0.4234	0.6070
	(202)	0.4365	0.4239	0.2973	0.4611
	(004)	0.2467	0.2457	0.2937	0.6218
	(220)	0.3882	0.3363	0.3219	0.3693

**Table 12:** Instrumented indentation properties of the samples TBC-1 and TBC-2 after creep test at 500 °C and 125 MPa.

Sample	Region Analyzed	HIT After Creep [MPa]	EIT After Creep [GPa]
TBC-1	Top coat	14,725 ± 3404	110 ± 16
	Substrate	6394 ± 517	93 ± 5
TBC-2	Top coat	9645 ± 1591	83 ± 10
	Substrate	5639 ± 748	89 ± 7

## Data Availability

The original contributions presented in this study are included in the article. Further inquiries can be directed to the corresponding author.

## Abbreviations

The following abbreviations are used in this manuscript:

APS	Air Plasma Spray
BC	Bond coat
EIT	Indentation elastic modulus
FWHM	Full width at half maximum
HIT	Indentation hardness
TBC	Thermal barrier coating
TTBC	Thick thermal barrier coating
TC	Top coat
TGO	Thermally grown oxide
SEM	Scanning electron microscopy
XRD	X-ray diffraction
YSZ	Yttria stabilized zirconia
